# Synthesis, characterization, and tuberculostatic activity of novel 2-(4-nitrobenzoyl)hydrazinecarbodithioic acid derivatives

**DOI:** 10.1007/s00706-011-0708-y

**Published:** 2012-01-24

**Authors:** Katarzyna Gobis, Henryk Foks, Ewa Augustynowicz-Kopec, Agnieszka Napiorkowska, Malgorzata Szczesio, Andrzej Olczak, Marek L. Glowka

**Affiliations:** 1 Department of Organic Chemistry, Medical University of Gdansk, 107 Gen. Hallera Str., 80-416 Gdansk, Poland; 2 Department of Microbiology, Institute of Tuberculosis and Pulmonary Diseases, Warsaw, Poland; 3 Institute of General and Ecological Chemistry, Technical University of Lodz, Lodz, Poland

**Keywords:** Drug research, 5-(4-Nitrophenyl)-1,3,4-oxadiazole-, Tuberculostatic activity, Crystal structure, Structure–activity relationship

## Abstract

**Abstract:**

A series of novel *S*-esters of 2-(4-nitrobenzoyl)hydrazinecarbodithioic acid and *S*,*S′*-diesters of (4-nitrobenzoyl)carbonohydrazonodithioic acid were synthesized by reaction of 4-nitrobenzohydrazide and *N*-methyl-4-nitrobenzohydrazide with carbon disulfide and alkyl halides in the presence of triethylamine. Novel 5-(4-nitrophenyl)-1,3,4-oxadiazoles were also obtained. The structures were confirmed by IR, NMR, and mass spectroscopy, and by elemental analysis. All the compounds obtained were screened in vitro for their tuberculostatic activity. Promising preliminary results were obtained for some of the compounds. The crystal structure of the most active compound was determined.

**Graphical abstract:**

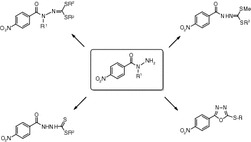

**Electronic supplementary material:**

The online version of this article (doi:10.1007/s00706-011-0708-y) contains supplementary material, which is available to authorized users.

## Introduction

Microorganisms of the *Mycobacterium* genus cause serious infectious diseases. Tuberculosis is one of these [1]. In recent years an increase in the incidence of that disease has been observed, and not only in developing countries [2]. Tuberculosis remains a global problem [3]. Failure of the therapy because of inadequately maintained treatment, abuse of antimicrobial drugs, and discontinuation of the treatment when symptoms disappear, favour the spread of the disease [4]. Improperly conducted therapy results in the appearance of resistant disease varieties, multidrug-resistant tuberculosis (MDR-TB), and extremely drug-resistant tuberculosis (XDR) [5]. The resistance of *Mycobacterium* strains is the cause of the increased risk of tuberculosis active form development and mortality in immunocompromised individuals, for example patients during immunosuppressive therapy or HIV-positive patients [6].

For many years isonicotinic acid hydrazide (isoniazid) has been one of the most commonly used anti-tuberculosis chemotherapeutics [7]. Unfortunately, mycobacteria rapidly develop resistance to this drug. Nevertheless, the isoniazid molecule is a leading structure for discovery of new chemotherapeutic agents [8]. In the course of this research many new compounds have been synthesized, for example hydrazones [9] and arylhydrazones [10]. Some of these have promising anti-tuberculosis activity [11].

**Fig. 1:**
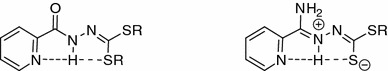
Intramolecular hydrogen bonds formed in molecules of *S*,*S′*-diesters of picolinoylcarbonohydrazonodithioic acid and amino(pyridine-2-yl)methylenecarbonohydrazonodithioic acid

**Fig. 2:**
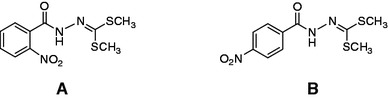
Structures of dimethyl 2-nitrobenzoylcarbonohydrazonodithioate (**a**) and dimethyl 4-nitrobenzoylcarbonohydrazonodithioate (**b**)

Our previous studies on the synthesis of new compounds with antituberculous activity helped formulate the hypothesis of the relationship between the tuberculostatic activity of compounds in the isoniazid analog group and the coplanar structure of their molecules. Among others *S*-methyl and *S*,*S′*-dimethyl amino(pyridin-2-ylmethylene)carbonohydrazonodithioates have been synthesized. These compounds had high tuberculostatic activity in vitro, with MIC (minimum concentrations inhibiting the growth of mycobacteria) of 3.13 μg/cm^3^ [12, 13]. Crystallographic studies performed on these derivatives indicated their molecules have a planar structure. The adoption of such a structure is a result of intramolecular hydrogen bond formation (Fig. 1). This condition is also met by the *S*-esters and *S*,*S′*-diesters of benzoylhydrazinecarbodithioic acids derived from benzohydrazides, especially those that have the appropriate substituents at C-2 position of the benzene ring. For some of the compounds in this group high tuberculostatic activity in vitro has been detected [14]. Unexpectedly, we found no correlation between the almost planar structure of a derivative with an NO_2_ group in C-2 position and its activity towards *Mycobacterium tuberculosis*. The structure of the most active compound with an NO_2_ group in C-4 position has no ability to form a hydrogen bond and adopt a planar structure (Fig. 2). That compound was the most active against H_37_Rv standard strain and sensitive 192 strain (MICs 3.1 μg/cm^3^). Its activity against resistant 210 strain was lower (MIC 25 μg/cm^3^).

In this work 2-(4-nitrobenzoyl)hydrazinecarbodithioates and (4-nitrobenzoyl)carbonohydrazonodithioates have been the synthesized for further verification of the hypothesis that planarity of hydrazinecarbodithioic acid derivatives correlates with their high tuberculostatic activity. We also expanded the synthesized group by preparing *S*,*S′*-diesters of *N*-methyl-4-nitrobenzoylcarbonohydrazonodithioic acid. In their structure, spatial hindrance by a methyl group attached to the nitrogen atom of the carbonohydrazonodithioic moiety prevents intramolecular hydrogen bond formation and, in accordance to our hypothesis, these compounds should have rather low tuberculostatic activity.

## Results and discussion

### Chemistry

**Scheme 1:**
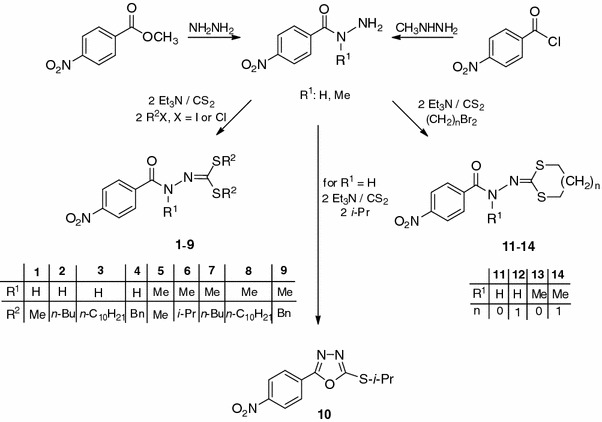

**Scheme 2:**
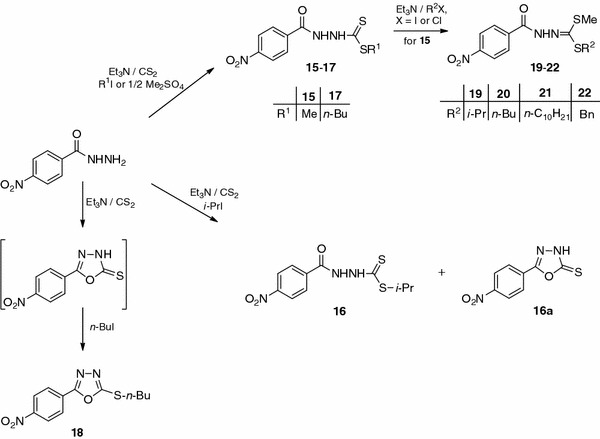

The synthetic route of the target compounds is outlined in Schemes 1 and 2. First, symmetrical *S*,*S′*-diesters of 4-nitrobenzoylocarbonohydrazonodithioic acid and *N*-methyl-4-nitrobenzoylcarbonohydrazonodithioic acid (**1**–**9**) were synthesized. Modification within the thioxyl group gave methyl, isopropyl, *n*-butyl, *n*-decyl, and benzyl *S*,*S′*-diesters. Substituents were chosen to differ substantially in size. Esters of 1,3-dithiolane and 1,3-dithiane with cyclic structures were also obtained (**11**–**14**, Scheme 1).

4-Nitrobenzoyl chloride and methyl 4-nitrobenzoate were the starting compounds for synthesis of all the derivatives obtained. Methyl 4-nitrobenzoate in dioxane under the action of hydrazine hydrate formed 4-nitrobenzohydrazide [15]. *N*-Methyl-4-nitrobenzohydrazide was obtained by reaction of 4-nitrobenzoyl chloride with a twofold excess of *N*-methylhydrazine in anhydrous dichloromethane [16]. Linear *S*,*S′*-diesters **1**–**9** and cyclic diesters **11**–**14** were then synthesized from 4-nitrobenzohydrazide and *N*-methyl-4-nitrobenzohydrazide. The reactions were conducted in methanol with a twofold excess of triethylamine (TEA). The products obtained by addition of carbon disulfide were alkylated with a twofold excess of the corresponding halides (methyl iodide, isopropyl iodide, *n*-butyl iodide, benzyl chloride, *n*-decyl iodide) or an equimolar amount of dihalides (1,2-dibromoethane, 1,3-dibromopropane). The reactions proceeded rapidly at room temperature and generally afforded the desired products in good yields (50–96%).

The interaction of 4-nitrobenzohydrazide with CS_2_ and isopropyl iodide in the presence of TEA did not lead to formation of diisopropyl 4-nitrobenzoylcarbonohydrazonodithioate, as is apparent from the absence of C=O and NH vibrations in the IR spectrum. In addition, in the ^1^H NMR spectrum a signal suggesting the presence of the NH group was not observed and results from integration were indicative of half the number of alkyl protons. By means of elemental analysis and mass spectrometry, the chemical formula C_11_H_11_N_3_O_3_S was suggested and MW 265 was determined; these correspond to cyclic 2-(isopropylthio)-5-(4-nitrophenyl)-1,3,4-oxadiazole (**10**). Such cyclization of methyl hydrazinecarbodithioates to 2-(methylthio)-1,3,4-oxadidiazoles in alkaline solution is known and has been already described [17, 18].

*S*-Esters **15**–**17** were also obtained from 4-nitrobenzohydrazide (Scheme 2). Reactions were carried out in a methanol solution of TEA using the corresponding iodides. For methyl ester **15** slightly less methyl iodide was used to prevent formation of the *S*,*S′*-diester, because of easy methylation [19]. In the synthesis of *S*-isopropyl ester **16** a side product **16a** was also formed, as is apparent from the absence of C=O vibrations and the presence of NH vibrations in the IR spectrum of that product. In the ^1^H NMR spectrum no signals of the isopropyl group were observed, and the singlet of the NH group was detected at 13.92 ppm. By means of elemental analysis and mass spectrometry, the chemical formula C_8_H_5_N_3_O_3_S was suggested and MW 223 was determined; these correspond to cyclic 5-(4-nitrophenyl)-1,3,4-oxadiazole-2(3*H*)-thione described elsewhere by Chau [20]. That conclusion was confirmed by the measured melting point of 204 °C. We have already reported cyclization of methyl hydrazinecarbodithioates to 1,3,4-oxadiazole-2(3*H*)-thiones [21]. 5-(4-Nitrophenyl)-1,3,4-oxadiazole-2(3*H*)-thione was unsuccessfully alkylated with isopropyl iodide in a methanol solution of TEA, affording 1,3,4-oxadiazole **10**. After 2 days of stirring, both at room temperature and under reflux, no 2-(isopropylthio)-5-(4-nitrophenyl)-1,3,4-oxadiazole was found in the reaction mixture. Alkylation in the presence of KOH, as strong base, in absolute ethanol was also attempted. The desired product was obtained in a yield of 25% only. The moderate efficiency of 5-phenyl and 5-pyridyl-1,3,4-oxadiazole-2(3*H*)thione alkylation (30–40%) with isopropyl iodide has been described by Muhi-eldeen and co-workers [22]. Other authors have conducted that type of alkylation in dioxane containing KOH, KI, Bu_4_NBr, and the corresponding halide [23, 24]. They also recommended addition of a catalytic amount of crown ether (18-crown-6 or 15-crown-5). It is, therefore, concluded that isopropyl 2-(4-nitrobenzoyl)hydrazinecarbodithioate (**16**) at room temperature readily cyclizes to 2-(isopropylthio)-5-(4-nitrophenyl)-1,3,4-oxadiazole (**10**), whereas 5-(4-nitrophenyl)-1,3,4-oxadiazol-2(3*H*)-thione undergoes alkylation with a secondary halide, i.e. isopropyl iodide, with unsatisfactory yield. Easy cyclization of *S*-isopropyl ester **16** prevents alkylation of the second sulfur atom in the synthesis of a diisopropyl *S*,*S′*-ester. This method seems more useful for obtaining 2-(isopropylthio)-5-(4-nitrophenyl)-1,3,4-oxadiazole (**10**).

In contrast with the 1,3,4-oxadiazole derivative **10** its butyl analogue **18** was obtained easily in methanol by addition of carbon disulfide to 4-nitrobenzohydrazide in the presence of TEA. The reaction was conducted at room temperature for 24 h. The resulting 1,3,4-oxadiazole-2(3*H*)-thione was then alkylated with *n*-butyl iodide without isolation.

Methyl *S*-ester **15** when treated with the respective halides in a methanol solution of TEA gave unsymmetrical *S*,*S′*-diesters **19**–**22** with different substituents at both sulfur atoms. Introduction of different substituents on sulfur atoms would provide a greater variety of compound structures and enable determination of the effect of substituent size on the tuberculostatic activity of these derivatives.

The newly synthesized compounds were characterized by IR, NMR, and mass spectroscopy, and elemental analysis. Results from spectral analysis were in accordance with the assigned structures. In the ^1^H NMR spectra of compounds **2**, **4**, **5**, **11**, and **13** the alkyl groups of the thioalkyl moieties were detected as separate signals. For example, for compound **5** two singlets of two methyl groups were detected at chemical shifts 2.32 and 2.49 ppm. This indicates the magnetic inequivalence of both groups. We have already observed a similar phenomenon for other dimethyl benzoylcarbonohydrazonodithioates [14].

### Tuberculostatic activity

The newly synthesized 4-nitrobenzoylcarbonohydrazonodithioates **2**–**9**, **11**–**14**, and **19**–**22**, 2-(4-nitrobenzoyl)hydrazinecarbodithioates **15**–**17**, and 5-(4-nitrophenyl)-1,3,4-oxadiazoles **10** and **18** were examined in vitro for their tuberculostatic activity against *M. tuberculosis* H_37_Rv strain and two “wild” strains isolated from tuberculosis patients: one (spec. 210) resistant to *p*-aminosalicylic acid (PAS), isonicotinic acid hydrazide (INH), etambutol (ETB), and rifampicine (RMP), and the another (spec. 192) fully sensitive to the administered tuberculostatics (Table 1).

**Table 1:** In-vitro tuberculostatic activity of synthesized compounds **1**–**3**, **5**–**7**, and **10**–**22**

No.	MIC/μg cm^−3^		
	H_37_Rv	Spec. 192	Spec. 210
**1**	25	12.5	50
**2**	50	50	50
**3**	50	50	50
**5**	50	50	50
**6**	50	50	50
**7**	100	100	100
**10**	12.5	6.2	25
**11**	50	25	50
**12**	50	50	50
**13**	100	50	50
**14**	50	50	50
**15**	50	25	50
**16**	50	25	50
**17**	50	25	50
**18**	6.2	6.2	25
**19**	25	12.5	25
**20**	25	6.2	50
**21**	25	12.5	50
**22**	25	12.5	50
INH	0.5	0.5	1.1
PZA	25	25	40

Minimum inhibitory concentrations for the bacterial strains were determined by the twofold serial dilution method for microdilution plates, and for the mycobacterial strains by the twofold classical test-tube method of successive dilution

INH, isoniazid; PZA, pyrazinamide

*M. tuberculosis* H_37_Rv, spec. 192, spec. 210

The results from measurement of tuberculostatic activity indicated that some of the title compounds had moderate activity against tested strains in vitro and were less active than isoniazid (INH) but of comparable activity to pyrazinamide (PZA), used as reference drugs. The MIC values ranged from 25 to 100 μg/cm^3^ for most of the compounds tested, from 0.5 to 1.1 μg/cm^3^ for INH, and from 25 to 40 μg/cm^3^ for PZA. There were no differences in sensitivity to the tested compounds between sensitive 192 and resistant 210 strain. Dimethyl 4-nitrobenzoylcarbonohydrazonodithioate (**1**) had the highest tuberculostatic activity in vitro among all the *S*-esters and symmetrical *S*,*S′*-diesters obtained. The MIC values for compound **1** were 25 (H_37_Rv), 12.5 (spec. 192), and 50 μg/cm^3^ (spec. 210). Compound **19**, i.e. isopropyl methyl ester, was the most active in the group of unsymmetrical *S*,*S′*-diesters. The MIC values determined for that compound were 12.5 and 25 μg/cm^3^. Given that all *S*,*S′*-symmetric diesters had rather weak tuberculostatic activity, the size of substituents at sulfur atoms seemed to be important for the activity—at least one should be small in volume. These results enabled inclusion of the obtained *S*-esters and *S*,*S′*-diesters of 4-nitrobenzoylcarbonohydrazonodithioic acid among the compounds with moderate tuberculostatic activity comparable with that of clinically used PZA (MICs 25–40 μg/cm^3^).

5-(4-Nitrophenyl)-1,3,4-oxadiazoles **10** and **18** had the highest activity among the group of compounds tested. The MIC values determined for these compounds were 6.2–12.5 μg/cm^3^ against sensitive strain 192 and the standard H_37_Rv strain, and 25 μg/cm^3^ against resistant 210 strain. These results indicated that 1,3,4-oxadiazoles have antimycobacterial activity in vitro, as reported in the literature [25, 26]. Therefore, further studies of this group of compounds seem appropriate.

### Molecular structure of compound **18** in the crystal

**Fig. 3:**
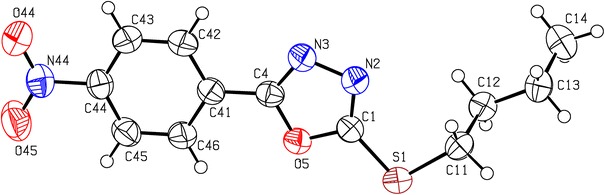
The molecular structure of **18**, showing the atom-numbering scheme. Displacement ellipsoids are drawn at the 50% probability level

X-ray diffraction was performed for the most active 1,3,4-oxadiazole **18**. Its molecular structure is shown in Fig. 3. Crystal data, details of data collections, reduction, and refinement are shown in Table 2.

**Table 2:** Crystal data and details of structure determination for compound **18**

Formula	C_12_H_13_N_3_ O_3_S
Formula weight	279.32
Crystal system	Monoclinic
Space group	*P*2_1_/*c*
*a*/Å *b*/Å *c*/Å *β/°*	14.6575(4) 10.0627(2) 8.8996(2) 92.665(1)
*V*/Å^3^	1311.22(5)
*Z*	4
Calculated density/g cm^−3^	1.415
F(000)	584
Radiation/Å	1.54178
No of reflections: measured, independent	13846, 2398
*R*_int_	0.029
Observed reflections [I > 2.0 σ(I)]	2237
No of parameters refined	173
*R*, _w_ *R*_2_, *S*	0.0467, 0.1406, 1.09
Min. and max. residual density/e Å^−3^	−0.20, 0.34

**Fig. 4:**
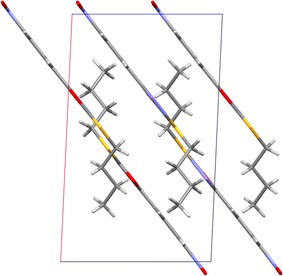
The molecular packing of **18** in the crystal, viewed perpendicular to the *b* axis

Partial stacking (with interplanar distance <3.5 Å) is observed for the planar fragments of the molecules, resulting in a semi-layered architecture of the crystal with butyl groups fitting between the layers (Fig. 4). The only other packing forces present in the structure are weak intermolecular H11B–C11···N3 contacts.

In summary, a series of novel *S*-esters and *S*,*S′*-diesters of 4-nitrobenzoylcarbonohydrazonodithioic acid and 5-(4-nitrophenyl)-1,3,4-oxadiazoles with different alkyl moieties on sulfur atoms were synthesized successfully. Unexpectedly, two cyclic products were obtained. 2-(Isopropylthio)-5-(4-nitrophenyl)-1,3,4-oxadiazole was obtained instead of the diisopropyl ester and 5-(4-nitrophenyl)-1,3,4-oxadaizole-2(3*H*)-thione was synthesized in addition to the *S*-isopropyl ester. Because of the easy cyclization, synthesis of 2-(butylthio)-5(4-nitrophenyl)-1,3,4-oxadiazole was conducted. The structures of the new compounds were confirmed by IR, NMR, and mass spectroscopy, and by elemental analysis. Their tuberculostatic activity was evaluated against *M. tuberculosis* H_37_Rv standard strain, sensitive strain 192, and 210 strain resistant to commonly administrated drugs (PAS, INH, ETB, RMP). MIC values were determined by use of the twofold serial dilution technique. The results showed that most of the synthesized derivatives had moderate tuberculostatic activity in vitro. The most active compounds were 1,3,4-oxadiazoles **10** and **18**. The MIC values determined for those compounds ranged from 6.2 to 25 μg/cm^3^. X-ray diffraction was performed for 1,3,4-oxadiazole **18**. These results enabled inclusion of the 5-(4-nitrophenyl)-1,3,4-oxadiazoles obtained among compounds with promising activity against Mtb.

## Experimental

Melting points were determined with a Boethius apparatus (Franz Küstner, Dresden, Germany). IR spectra of KBr pellets or paraffin oil suspensions were acquired on a Satellite FT-IR spectrophotometer (Mattson Instruments, Madison, WI, USA). ^1^H and ^13^C NMR spectra were acquired in CDCl_3_ and DMSO-*d*_6_ on Varian Gemini (200 MHz) and Varian Unity Plus (500 MHz) instruments (Varian, Palo Alto, CA, USA). Results from elemental analysis (% C, H, N) were in agreement with calculated values within the ±0.3% range for all the compounds. Mass spectra for compounds **2**, **10**, **12**, **16**, **16a**, **18**, and **19** were acquired on a Biosystem Qstar XL MS/MS spectrometer with electrospray ionization (70 eV). Gravity column chromatography for compounds **2**–**4**, **7**, **8**, and **17** was performed on Fluka silica gel 60 of particle size 0.063–0.2 mm and 70–230 mesh ASTM (Sigma–Aldrich Chemie, Steinheim, Germany). Synthesis of 4-nitrobenzohydrazide and *N*-methyl-4-nitrobenzohydrazide is described elsewhere [15, 16]; reaction yields and compound characteristics were found to be identical with those reported. 4-Nitrobenzoic acid 2-[bis(methylthio)methylene]hydrazide (**1**) was synthesized by the method reported recently [14]; reaction details and compound characteristics corresponded with literature data.

### General procedure for synthesis of symmetrical S,S′-diesters **1**–**9**

4**-**Nitrobenzohydrazide or *N*-methyl-4-nitrobenzohydrazide (2.5 mmol) was suspended in 5 cm^3^ methanol and 0.87 cm^3^ TEA (6.25 mmol) and 0.23 cm^3^ CS_2_ (3.75 mmol) were added. The mixture was stirred at room temperature for 1 h. Then 6.25 mmol of the respective halide was added and the mixture was stirred for another 2 h. Then 20 g ice was added, and the precipitate of the dimethyl ester (**1**, **5**) was isolated by filtration, dried, and recrystallized from methanol. All other products were extracted from the methanol–water solution with chloroform (3 × 10 cm^3^), the extract was dried with MgSO_4_, and the solvent was evaporated under vacuum. Crude products **6**–**9** were treated with dry diethyl ether, isolated by filtration, dried, and recrystallized from methanol. Products **2**–**4** were purified by column chromatography.

#### 4-*Nitrobenzoic acid* 2-*[bis(butylthio)methylene]hydrazide* (**2**, C_16_H_23_N_3_O_3_S_2_)

This product was purified by column chromatography with chloroform–cyclohexane (2:1) as eluent. The compound was obtained as an orange solid in 75% yield (0.69 g). M.p.: 58–60 °C; IR (KBr):$\bar{v}$ = 3,253 (*ν* N–H), 2,958, 2,928, 2,873 (*ν* C–H), 1,648 (*ν* C=O), 1,545, 1,527 (*ν* NO_2_), 1,484 (*ν* C=C), 1,341, 1,329 (*δ* C–H), 850 (*ν* C–N), 707 (γ C–C) cm^−1^; ^1^H NMR (200 MHz, CDCl_3_): *δ* = 0.82 (t, 3H, CH_3_, *J* = 7 Hz), 0.94 (t, 3H, CH_3_, *J* = 7 Hz), 1.26–1.38 (m, 2H, CH_2_), 1.42–1.49 (m, 4H, 2 CH_2_), 1.62–1.66 (m, 2H, CH_2_), 2.90 (t, 2H, SCH_2_, *J* = 7.3 Hz), 3.03 (t, 2H, SCH_2_, *J* = 7.3 Hz), 8.10 (d, 2H, Ph, *J* = 8.8 Hz), 8.17 (d, 2H, Ph, *J* = 8.8 Hz), 9.12 (s, 1H, NH) ppm; ^13^C NMR (50 MHz, CDCl_3_): *δ* = 14.03 (CH_3_), 22.41 (CH_2_), 31.21 (SCH_2_), 32.73 (CH_2_), 123.94 (Ph), 128.93 (Ph), 142.75 (Ph-CO), 149.34 (Ph-NO_2_), 157.25 (C=N), 168.75 (C=O) ppm; MS (70 eV): *m*/*z* = 369.

#### *4*-*Nitrobenzoic acid 2*-*[bis(decylthio)methylene]hydrazide* (**3**, C_28_H_47_N_3_O_3_S_2_)

This product was purified by column chromatography with chloroform–cyclohexane (2:1) as eluent. The compound was obtained as a pale yellow oil in 73% yield (0.98 g). IR:$\bar{v}$ = 3,280 (*ν* N–H), 2,925, 2,853 (*ν* C–H), 1,673 (*ν* C=O), 1,602 (*ν* C=C), 1,527 (*ν* NO_2_), 1,467 (*ν* C=C), 1,341 (*δ* C–H), 1,268 (*ν* C–N), 865, 849 (*ν* C–N), 715 (γ C–C) cm^−1^; ^1^H NMR (200 MHz, CDCl_3_): *δ* = 0.88 (t, 6H, 2 CH_3_, *J* = 6 Hz), 1.26–1.44 (m, 28H, 14 CH_2_), 1.56–1.76 (m, 4H, 2 CH_2_), 2.75–3.21 (m, 4H, 2 SCH_2_), 7.98 (d, 2H, Ph, *J* = 8.7 Hz), 8.22–8.35 (m, 2H, Ph), 9.68 (brs, 1H, NH) ppm; ^13^C NMR (50 MHz, CDCl_3_): *δ* = 14.27 (CH_3_), 21.98 (CH_2_), 26.53 (SCH_2_), 27.89 (CH_2_), 28.05 (CH_2_), 28.99 (CH_2_), 23.31 (CH_2_), 30.98 (CH_2_), 31.67 (CH_2_), 122.97 (Ph), 127.56 (Ph), 137.34 (Ph-CO), 149.78 (Ph-NO_2_), 162.28 (C=N), 169.60 (C=O) ppm.

#### *4*-*Nitrobenzoic acid 2*-*[bis(phenylmethylthio)methylene]hydrazide* (**4**, C_21_H_17_N_3_O_3_S_2_)

This product was purified by column chromatography with chloroform as eluent. The compound was obtained as a red solid in 50% yield (0.53 g). M.p.: 159–160 °C; IR (KBr):$\bar{v}$ = 3,289 (*ν* N–H), 2,924, 2,853 (*ν* C–H), 1,689 (*ν* C=O), 1,552 (*ν* NO_2_), 1,522, 1,497, 1,478 (*ν* C=C), 1,337 (*δ* C–H), 849 (*ν* C–N), 711, 698 (γ C–C) cm^−1^; ^1^H NMR (200 MHz, CDCl_3_): *δ* = 4.08 (s, 2H, SCH_2_), 4.38 (s, 2H, SCH_2_), 7.03–7.37 (m, 8H, Ph), 7.52–7.67 (m, 4H, Ph), 8.24 (d, 2H, Ph, *J* = 8.3 Hz), 9.45 (s, 1H, NH) ppm; ^13^C NMR (50 MHz, DMSO-*d*_6_): *δ* = 36.36 (SCH_2_), 37.29 (SCH_2_), 122.98 (Ph), 127.34 (Ph), 127.41 (Ph), 128.67 (Ph), 128.93 (Ph), 129.27 (Ph), 129.36 (Ph), 129.63 (Ph), 137.66 (Ph-CO), 146.05 (Ph-NO_2_), 163.85 (C=N), 174.03 (C=O) ppm.

#### *4*-*Nitrobenzoic acid 2*-*[bis(methylthio)methylene]*-*1*-*methylhydrazide* (**5**, C_11_H_13_N_3_O_3_S_2_)

This compound was obtained as yellow needles in 62% yield (0.46 g). M.p.: 98–100 °C; IR (KBr):$\bar{v}$ = 3,068, 3,046, 3,007 (*ν* C–H), 1,642 (*ν* C=O), 1,598 (*ν* C=C), 1,518 (*ν* NO_2_), 1,415 (*ν* C=C), 1,348 (*δ* C–H), 1,023, 950 (*δ* C–H), 870, 852 (*ν* C–N), 718 (γ C–C) cm^−1^; ^1^H NMR (200 MHz, CDCl_3_): *δ* = 2.32 (s, 3H, SCH_3_), 2.49 (s, 3H, SCH_3_), 3.30 (s, 3H, NCH_3_), 7.71 (d, 2H, Ph, *J* = 8.9 Hz), 8.18 (d, 2H, Ph, *J* = 8.7 Hz) ppm; ^13^C NMR (50 MHz, DMSO-*d*_6_): *δ* = 13.95 (SCH_3_), 15.18 (SCH_3_), 35.20 (NCH_3_), 123.31 (Ph), 129.01 (Ph), 141.81 (Ph-CO), 148.26 (Ph-NO_2_), 166.79 (C=N), 176. 85 (C=O) ppm.

#### *4*-*Nitrobenzoic acid 2*-*[bis[(1*-*methylethyl)thio]methylene]*-*1*-*methylhydrazide* (**6**, C_15_H_21_N_3_O_3_S_2_)

This compound was obtained as a pale yellow solid in 27% yield (0.24 g). M.p.: 89–90 °C; IR (KBr):$\bar{v}$ = 2,961, 2,927, 2,865 (*ν* C–H), 1,639 (*ν* C=O), 1,600 (*ν* C=C), 1,521 (NO_2_), 1,497 (*ν* C=C), 1,352 (*δ* C–H), 989, 866 (*ν* C–N), 723 (γ C–C) cm^−1^; ^1^H NMR (200 MHz, CDCl_3_): *δ* = 1.14 (d, 6H, 2 CH_3_, *J* = 6 Hz), 1.38 (d, 6H, 2 CH_3_, *J* = 6.7 Hz), 3.27 (s, 3H, NCH_3_), 3.65–3.72 (m, 2H, 2 SCH), 7.68 (d, 2H, Ph, *J* = 8.8 Hz), 8.18 (d, 2H, Ph, *J* = 8.4 Hz) ppm; ^13^C NMR (50 MHz, CDCl_3_): *δ* = 21.34 (CH_3_), 23.94 (SCH), 40.95 (NCH_3_), 122.81 (Ph), 124.09 (Ph), 128.35 (Ph), 130.71 (Ph), 139.23 (Ph-CO), 148.27 (Ph-NO_2_), 159.93 (C=N), 172.71 (C=O) ppm.

#### *4*-*Nitrobenzoic acid 2*-*[bis(butylthio)methylene]*-*1*-*methylhydrazide* (**7**, C_17_H_25_N_3_O_3_S_2_)

This product was purified by column chromatography with chloroform as eluent. The compound was obtained as a light yellow oil in 61% yield (0.58 g). IR:$\bar{v}$ = 2,959, 2,931, 2,872 (*ν* C–H), 1,643 (*ν* C=O), 1,600 (*ν* C=C), 1,523 (*ν* NO_2_), 1,348 (*δ* C–H), 1,221 (*ν* C–C), 1,045, 992 (*δ* C–H), 863 (*ν* C–N), 719 (γ C–C) cm^−1^; ^1^H NMR (200 MHz, CDCl_3_): *δ* = 0.77–0.98 (m, 6H, 2 CH_3_), 1.21–1.73 (m, 8H, 4 CH_2_), 2.86–3.00 (m, 4H, 2 SCH_2_), 3.29 (s, 3H, NCH_3_), 7.68 (d, 2H, Ph, *J* = 9 Hz), 8.19 (d, 2H, Ph, *J* = 8.6 Hz) ppm; ^13^C NMR (50 MHz, CDCl_3_): *δ* = 13.90 (CH_3_), 13.96 (CH_3_), 22.27 (CH_2_), 31.63 (CH_2_), 31.91 (SCH_2_), 35.14 (NCH_3_), 123.32 (Ph), 129.10 (Ph), 142.61 (Ph-CO), 148.81 (Ph-NO_2_), 163.96 (C=N), 176.11 (C=O) ppm.

#### *4*-*Nitrobenzoic acid 2*-*[bis(decylthio)methylene]*-*1*-*methylhydrazide* (**8**, C_29_H_49_N_3_O_3_S_2_)

This product was purified by column chromatography with chloroform as eluent. The compound was obtained as a yellow oil in 96% yield (1.32 g). IR:$\bar{v}$ = 2,924, 2,853 (*ν* C–H), 1,648 (*ν* C=O), 1,601 (*ν* C=C), 1,525 (*ν* NO_2_), 1,491, 1,464 (*ν* C=C), 1,347 (*δ* C–H), 1,220 (*ν* C–C), 1,071, 1,043, 994 (*δ* C–H), 862, 850 (*ν* C–N), 719 (γ C–C) cm^−1^; ^1^H NMR (200 MHz, CDCl_3_): *δ* = 0.85–0.92 (m, 6H, 2 CH_3_), 1.12–1.27 (m, 32H, 16 CH_2_), 2.83–2.90 (m, 4H, 2 SCH_2_), 3.29 (s, 3H, NCH_3_), 7.68 (d, 2H, Ph, *J* = 8.9 Hz), 8.18 (d, 2H, Ph, *J* = 8.7 Hz) ppm; ^13^C NMR (50 MHz, CDCl_3_): *δ* = 14.38 (CH_3_), 22.85 (CH_2_), 27.58 (SCH_2_), 28.49 (CH_2_), 29.31 (CH_2_), 29.86 (CH_2_), 30.05 (CH_2_), 31.97 (CH_2_), 32.11 (CH_2_), 39.86 (N-CH_2_), 122.79 (Ph), 128.52 (Ph), 142.61 (Ph-CO), 149. 76 (Ph-NO_2_), 162.31 (C=N), 175.89 (C=O) ppm.

#### *4*-*Nitrobenzoic acid 2*-*[bis(phenylmethylthio)methylene]*-*1*-*methylhydrazide* (**9**, C_23_H_21_N_3_O_3_S_2_)

This compound was obtained as colorless crystals in 65% yield (0.73 g). M.p.: 78–79 °C; IR (KBr):$\bar{v}$ = 3,029, 2,983, 2,936 (*ν* C–H), 1,634 (*ν* C=O), 1,601 (*ν* C=C), 1,520 (*ν* NO_2_), 1,481 (*ν* C=C), 1,354, 1,340 (*δ* C–H), 994 (*ν* C–N), 711 (γ C–C) cm^−1^; ^1^H NMR (500 MHz, CDCl_3_): *δ* = 3.30 (s, 3H, NCH_3_), 4.25 (s, 4H, 2 SCH_2_), 7.28–7.38 (m, 10H, 2 Ph), 7.59 (d, 2H, Ph, *J* = 7.3 Hz), 8.04–8.08 (m, 2H, Ph) ppm; ^13^C NMR (50 MHz, CDCl_3_): *δ* = 36.00 (NCH_3_), 36.59 (SCH_2_), 122.85 (Ph), 127.70 (Ph), 128.15 (Ph), 128.59 (Ph), 128.78 (Ph), 128.88 (Ph), 129.00 (Ph), 134.82 (Ph-CH_2_), 141.63 (Ph-CO), 149.51 (Ph-NO_2_), 161.83 (C=N), 173.21 (C=O) ppm.

#### *2*-*[(1*-*Methylethyl)thio]*-*5*-*(4*-*nitrophenyl)*-*1,3,4*-*oxadiazole* (**10**, C_11_H_11_N_3_O_3_S)

Method A: the compound was synthesized from 0.45 g 4-nitrobenzohydrazide (2.5 mmol), 0.87 cm^3^ TEA (6.25 mmol), 0.23 cm^3^ CS_2_ (3.75 mmol), and 0.62 cm^3^ isopropyl iodide (6.25 mmol) by the method described for symmetrical diesters **1**–**9**. The reaction mixture was stirred for 5 days. Ice (20 g) was then added, the solution was extracted with chloroform (3 × 10 cm^3^), the extract was dried with MgSO_4_, and the solvent was evaporated under vacuum. The crude product was treated with dry diethyl ether, isolated by filtration, dried, and recrystallized from methanol to give yellow flakes in 63% yield (0.42 g).

Method B: 0.456 g 5-(4-nitrophenyl)-1,3,4-oxadiazole-2(3*H*)-thione (2 mmol) was suspended in 35 cm^3^ anhydrous ethanol and 0.12 g KOH (2 mmol) was added and the solution was heated under reflux for 30 min. Then 0.25 cm^3^ isopropyl iodide (2.5 mmol) was added dropwise and the mixture was heated under reflux for another 5 h. The reaction mixture was cooled, filtered, and the filtrate was poured on 100 g ice. The precipitate was isolated by filtration, dried, and recrystallized to give yellow flakes in 25% yield (0.132 g). M.p.: 91–93 °C; IR:$\bar{v}$ = 2,974, 2,924, 2,870 (*ν* C–H), 1,606 (*ν* C=C), 1521 (*ν* NO_2_), 1,465 (*ν* C=C), 1,347, 1,070 (*δ* C–H), 863, 850 (*ν* C–N), 726, 705 (γ C–C) cm^−1^; ^1^H NMR (200 MHz, CDCl_3_): *δ* = 1.55 (d, 6H, 2 CH_3_, *J* = 6.7 Hz), 3.95–4.09 (m, 2H, 2 SCH), 8.19 (d, 2H, Ph, *J* = 8.9 Hz), 8.35 (d, 2H, Ph, *J* = 8.9 Hz) ppm; ^13^C NMR (50 MHz, CDCl_3_): *δ* = 23.78 (CH_3_), 39.81 (SCH), 124.85 (Ph), 127.98 (Ph), 129.64 (Ph-1,3,4-oxadiazole), 149.90 (Ph-NO_2_), 164.21 (1,3,4-oxadiazole-Ph), 166.29 (1,3,4-oxadiazole-S) ppm; MS (70 eV): *m*/*z* = 265.

### General procedure for synthesis of cyclic diesters **11**–**14**

4-Nitrobenzohydrazide or *N*-methyl-4-nitrobenzohydrazide (2.5 mmol) was suspended in 5 cm^3^ methanol and 0.87 cm^3^ TEA (6.25 mmol) and 0.23 cm^3^ CS_2_ (3.75 mmol) were added. The mixture was stirred for 1 h at room temperature. Then 3.75 mmol 1,2-dibromoethane or 1,3-dibromopropane was added and the mixture was stirred for another 2 h. Ice (20 g) was then added. The precipitates of products **11** and **12** were isolated by filtration, dried, and recrystallized from dioxane–water (1:1). The solutions of products **13** and **14** were extracted with chloroform (3 × 10 cm^3^), the extracts were dried with MgSO_4_, and the solvent was evaporated under vacuum. The crude products were treated with dry diethyl ether, isolated by filtration, dried, and recrystallized from methanol.

#### *4*-*Nitrobenzoic acid 2*-*(1,3*-*dithiolan*-*2*-*ylidene)hydrazide* (**11**, C_10_H_9_N_3_O_3_S_2_)

This compound was obtained as pale yellow crystals in 84% yield (0.59 g). M.p.: 199–200 °C; IR (KBr):$\bar{v}$ = 3,228 (*ν* N–H), 2,980, 2,936 (*ν* C–H), 1,646 (*ν* C=O), 1,599 (*ν* C=C), 1,564 (*ν* C=C), 1,511 (*ν* NO_2_), 1,343 (*δ* C–H), 1,285 (*ν* C–C), 1,040 (*δ* C–H), 849 (*ν* C–N), 721 (γ C–C) cm^−1^; ^1^H NMR (200 MHz, CDCl_3_): *δ* = 3.50–3.56 (m, 2H, SCH_2_), 3.67–3.73 (m, 2H, SCH_2_), 8.00 (d, 2H, Ph, *J* = 8.8 Hz), 8.31 (d, 2H, Ph, *J* = 8.1 Hz), 9.15 (s, 1H, NH) ppm; ^13^C NMR (50 MHz, DMSO-*d*_*6*_): *δ* = 35.91 (SCH_2_), 123.81 (Ph), 129.37 (Ph), 139.35 (Ph-CO), 149.37 (Ph-NO_2_), 161.33 (C=N), 173.05 (C=O) ppm.

#### *4*-*Nitrobenzoic acid 2*-*(1,3*-*dithian*-*2*-*ylidene)hydrazide* (**12**, C_11_H_11_N_3_O_3_S_2_)

This compound was obtained as a yellow solid in 39% yield (0.29 g). M.p.: 140–142 °C; IR (KBr):$\bar{v}$ = 3,372 (*ν* N–H), 2,976 (*ν* C–H), 1,660 (*ν* C=O), 1,599 (*ν* C=C), 1,520 (*ν* NO_2_), 1,343 (*δ* C–H), 1,281 (*ν* C–C), 1,108, 1,052 (*δ* C–H), 849 (*ν* C–N), 715 (γ C–C) cm^−1^; ^1^H NMR (200 MHz, CDCl_3_): *δ* = 2.21–2.34 (m, 2H, CH_2_), 3.10–3.27 (m, 4H, 2 SCH_2_), 8.00 (d, 2H, Ph, *J* = 8.7 Hz), 8.30 (d, 2H, Ph, *J* = 8.1 Hz), 8.92 (s, 1H, NH) ppm; ^13^C NMR (50 MHz, CDCl_3_): *δ* = 36.11 (CH_2_), 39.36 (SCH_2_), 124.41 (Ph), 128.93 (Ph), 139.04 (Ph-CO), 152.11 (Ph-NO_2_), 161. 25 (C=N), 169.15 (C=O) ppm; MS (70 eV): *m*/*z* = 297.

#### *4*-*Nitrobenzoic acid 2*-*(1,3*-*dithiolan*-*2*-*ylidene)*-*1*-*methylhydrazide* (**13**, C_11_H_11_N_3_O_3_S_2_)

This compound was obtained as colorless crystals in 93% yield (0.69 g). M.p.: 158–159 °C; IR (KBr):$\bar{v}$ = 2,974, 2,936 (*ν* C–H), 1,645 (*ν* C=O), 1,601 (*ν* C=C), 1,538, 1,513 (*ν* NO_2_), 1,353 (*δ* C–H), 985 (*ν* C–N), 862 (γ C–C) cm^−1^; ^1^H NMR (500 MHz, CDCl_3_): *δ* = 3.37 (s, 3H, NCH_3_), 3.51 (t, 2H, SCH_2_, *J* = 5.9 Hz), 3.58 (t, 2H, SCH_2_, *J* = 5.4 Hz), 7.73 (d, 2H, Ph, *J* = 8.8 Hz), 8.22 (d, 2H, Ph, *J* = 8.8 Hz) ppm; ^13^C NMR (125 MHz, CDCl_3_): *δ* = 34.73 (NCH_3_), 36.54 (SCH_2_), 123.44 (Ph), 129.12 (Ph), 141.70 (Ph-CO), 148.37 (Ph-NO_2_), 166. 55 (C=N), 175.39 (C=O) ppm.

#### *4*-*Nitrobenzoic acid 2*-*(1,3*-*dithian*-*2*-*ylidene)*-*1*-*methylhydrazide* (**14**, C_12_H_13_N_3_O_3_S_2_)

This compound was obtained as pale yellow crystals in 75% yield (0.58 g). M.p.: 87–89 °C; IR (KBr):$\bar{v}$ = 2,927 (*ν* C–H), 1,634 (*ν* C=O), 1,597 (*ν* C=C), 1,518 (*ν* NO_2_), 1,351 (*δ* C–H), 853 (*ν* C–N), 713 (γ C–C) cm^−1^; ^1^H NMR (500 MHz, CDCl_3_): *δ* = 2.20–2.22 (m, 2H, CH_2_), 3.07 (s, 3H, NCH_3_), 3.24–3.39 (m, 4H, SCH_2_), 7.75 (d, 2H, Ph, *J* = 8.8 Hz), 8.23 (d, 2H, Ph, *J* = 8.3 Hz) ppm; ^13^C NMR (125 MHz, CDCl_3_): *δ* = 33.98 (CH_2_), 36.07 (SCH_2_), 38.99 (NCH_3_), 122.95 (Ph), 128.43 (Ph), 142.67 (Ph-CO), 149.78 (Ph-NO_2_), 159.93 (C=N), 167.83 (C=O) ppm.

#### *4*-*Nitrobenzoic acid 2*-*[(methylthio)thioxomethyl]hydrazide* (**15**, C_9_H_9_N_3_O_3_S_2_)

4-Nitrobenzohydrazide (0.45 g, 2.5 mmol) was suspended in 10 cm^3^ methanol and 0.348 cm^3^ TEA (2.5 mmol) and 0.151 cm^3^ CS_2_ (2.5 mmol) were added. The mixture was stirred for 1 h at room temperature and 0.12 cm^3^ methyl iodide (2 mmol) was added. The mixture was stirred for another 15 min and 20 g ice was added. A small amount of precipitate was removed by filtration and the filtrate was acidified with 0.183 cm^3^ acetic acid (2.5 mmol). The precipitate of the monoester was isolated by filtration, dried, and recrystallized from methanol to give a white solid in 46% yield (0.31 g). M.p.: 186–188 °C; IR (KBr):$\bar{v}$ = 3,285, 3,152 (*ν* N–H), 2,842 (*ν* C–H), 1,650 (*ν* C=O), 1,598 (*ν* C=C), 1,525 (*ν* NO_2_), 1,345 (*δ* C–H), 1,265 (*ν* C–N), 985 (*δ* C–H), 865 (*ν* C–N), 728 (γ C–C) cm^−1^; ^1^H NMR (200 MHz, CDCl_3_): *δ* = 2.73 (s, 3H, SCH_3_), 8.05 (d, 2H, Ph, *J* = 8.8 Hz), 8.37 (d, 2H, Ph, *J* = 8.8 Hz), 10.05 (s, 1H, NH), 10.55 (s, 1H, NH) ppm; ^13^C NMR (50 MHz, CDCl_3_): *δ* = 19.21 (SCH_3_), 122.92 (Ph), 127.99 (Ph), 139.88 (Ph-CO), 148.51 (Ph-NO_2_), 165.78 (C=O), 202.64 (C=S) ppm.

### Synthesis of 4-nitrobenzoic acid 2-[[(1-methylethyl)thio]thioxomethyl]hydrazide (**16**) and 5-(4-nitrophenyl)-1,3,4-oxadiazole-2(3H)-thione (**16a**)

4-Nitrobenzohydrazide (0.45 g, 2.5 mmol) was suspended in 10 cm^3^ methanol and 0.383 cm^3^ TEA (2.75 mmol) and 0.227 cm^3^ CS_2_ (3.75 mmol) were added. The mixture was stirred for 1 h and 0.250 cm^3^ isopropyl iodide (2.5 mmol) was added. The mixture was stirred for another 48 h and 20 g ice was added. The precipitate of monoester **16** was isolated by filtration, dried, and recrystallized from methanol–water (1:1). The filtrate was acidified with 0.183 cm^3^ acetic acid (2.5 mmol). The precipitate of 1,3,4-oxadiazole-2-thione **16a** was isolated by filtration, dried, and recrystallized from methanol.

#### *4*-*Nitrobenzoic acid 2*-*[[(1*-*methylethyl)thio]thioxomethyl]hydrazide* (**16**, C_11_H_23_N_3_O_3_S_2_)

This compound was obtained as a pale beige solid in 19% yield (0.14 g). M.p.: 172–174 °C; IR (KBr):$\bar{v}$ = 3,182 (*ν* N–H), 2,968, 2,922 (*ν* C–H), 1,672 (*ν* C=O), 1,601 (*ν* C=C), 1,524 (*ν* NO_2_), 1,324 (*δ* C–H), 1,283 (*ν* C–N), 1,035 (*δ* C–H), 864 (*ν* C–N), 718 (γ C–C) cm^−1^; ^1^H NMR (200 MHz, CDCl_3_): *δ* = 1.46 (d, 6H, 2 CH_3_, *J* = 6.9 Hz), 4.03–4.17 (m, 1H, SCH), 8.05 (d, 2H, Ph, *J* = 8.8 Hz), 8.37 (d, 2H, Ph, *J* = 8.8 Hz), 10.21 (s, 1H, NH), 10.67 (s, 1H, NH) ppm; ^13^C NMR (50 MHz, DMSO-*d*_*6*_): *δ* = 22.45 (CH_3_), 39.28 (SCH), 123.81 (Ph), 129.08 (Ph), 137.28 (Ph-CO), 149.72 (Ph-NO_2_), 163.74 (C=O), 202.31 (C=S) ppm; MS (70 eV): *m*/*z* = 299.

#### *5(4*-*Nitrophenyl)*-*1,3,4*-*oxadiazole*-*2(3H)*-*thione* (**16a**)

This compound was obtained as pale yellow needles in 55% yield (0.31 g). M.p.: 203–205 °C; the ^1^H NMR spectrum corresponded to the literature spectrum [20].

#### *4*-*Nitrobenzoic acid 2*-*[(butylthio)thioxomethyl]hydrazide* (**17**, C_12_H_15_N_3_O_3_S_2_)

4-Nitrobenzohydrazide (0.45 g, 2.5 mmol) was suspended in 10 cm^3^ methanol and 0.383 cm^3^ TEA (2.75 mmol) and 0.227 cm^3^ CS_2_ (3.75 mmol) were added. The mixture was stirred for 1 h at room temperature and 0.285 cm^3^ butyl iodide (2.5 mmol) was added. The mixture was stirred for 45 min and the solvent was evaporated under vacuum. Then 20 g ice was added to the residue. The solution was acidified with 0.183 cm^3^ acetic acid (2.5 mmol) and extracted with chloroform (3 × 10 cm^3^). The chloroform fractions were combined, dried with MgSO_4_, and the solvent was evaporated under vacuum. Components of the mixture were separated and purified by column chromatography with chloroform as eluent. The product was obtained as a pale yellow solid in 29% yield (0.23 g). M.p.: 110–112 °C; IR (KBr):$\bar{v}$ = 3,151 (*ν* N–H), 2,960, 2,930 (*ν* C–H), 1,665 (*ν* C=O), 1,600 (*ν* C=C), 1,524 (*ν* NO_2_), 1,439 (*ν* C=C), 1,340 (*δ* C–H), 1,292 (*ν* C–N), 708 (γ C–C) cm^−1^; ^1^H NMR (200 MHz, DMSO-*d*_6_): *δ* = 0.84–0.94 (m, 3H, CH_3_), 1.30–1.44 (m, 2H, CH_2_), 1.55–1.66 (m, 2H, CH_2_), 3.13–3.29 (m, 2H, SCH_2_), 8.10 (d, 2H, Ph, *J* = 8.7 Hz), 8.35–8.41 (m, 2H, Ph), 11.27 (s, 1H, NH), 11.61 (s, 1H, NH) ppm; ^13^C NMR (50 MHz, DMSO-*d*_*6*_): *δ* = 13.79 (CH_3_), 21.76 (CH_2_), 30.66 (CH_2_), 33.56 (SCH_2_), 124.23 (Ph), 129.33 (Ph), 137.58 (Ph-CO), 149.96 (Ph-NO_2_), 164.10 (C=O), 203.45 (C=S) ppm.

#### *2*-*(Butylthio)*-*5*-*(4*-*nitrophenyl)*-*1,3,4*-*oxadiazole* (**18**, C_12_H_13_N_3_O_3_S)

4-Nitrobenzohydrazide (0.91 g, 5 mmol) was suspended in 30 cm^3^ methanol, 0.836 cm^3^ TEA (6 mmol) and 0.453 cm^3^ CS_2_ (7.5 mmol) were added, and the mixture was stirred overnight at room temperature. The precipitate of an unidentified side product was removed by filtration and 0.569 cm^3^ butyl iodide (5 mmol) was added to the filtrate. The mixture was stirred for 3 h at room temperature, and the precipitate was isolated by filtration, dried, and recrystallized from methanol to give bright needles in 82% yield (1.15 g). M.p.: 99–100 °C; IR (KBr):$\bar{v}$ = 2,960, 2,943 (*ν* C–H), 1,605, 1,560 (*ν* C=C), 1,516 (*ν* NO_2_), 1,455 (*ν* C=C), 1,344, 1,185 (*δ* *δ* C–H), 853 (*ν* C–N), 706 (γ C–C) cm^−1^; ^1^H NMR (200 MHz, CDCl_3_): *δ* = 0.98 (t, 3H, CH_3_, *J* = 7.3 Hz), 1.46–1.61 (m, 2H, CH_2_), 1.78–1.94 (m, 2H, CH_2_), 3.35 (t, 2H, SCH_2_, *J* = 7.3 Hz), 8.20 (d, 2H, Ph, *J* = 9 Hz), 8.37 (d, 2H, Ph, *J* = 9 Hz) ppm; ^13^C NMR (50 MHz, CDCl_3_): *δ* = 13.62 (CH_3_), 21.33 (CH_2_), 31.24 (CH_2_), 32.10 (SCH_2_), 124.85 (Ph), 127.97 (Ph), 128.87 (Ph-1,3,4-oxadiazole), 149.32 (Ph-NO_2_), 163.96 (1,3,4-oxadiazole-Ph), 165.65 (1,3,4-oxadiazole-S) ppm; MS (70 eV): *m*/*z* = 279.

### General procedure for the synthesis of unsymmetrical S,S′-diesters **19**–**22**

Methyl 2-(4-nitrobenzoyl)hydrazinecarbodithioate (**15**, 0.35 g, 1.3 mmol) was dissolved in 5 cm^3^ methanol and 0.272 cm^3^ TEA (1.95 mmol) and 1.95 mmol of the respective halide were added. The mixture was stirred for 1 h at room temperature. In the reaction with isopropyl iodide (product **19**) the reaction time was prolonged to 24 h. The mixture was then cooled in an ice bath, and the precipitate was isolated by filtration, dried, and recrystallized from methanol.

#### *4*-*Nitrobenzoic acid 2*-*[[(1*-*methylethyl)thio](methylthio)methylene]hydrazide* (**19**, C_12_H_15_N_3_O_3_S_2_)

This compound was obtained as a light yellow solid in 32% yield (0.13 g). M.p.: 107–108 °C; IR (KBr):$\bar{v}$ = 3,163 (*ν* N–H), 2,961, 2,923 (*ν* C–H), 1,667, 1,643 (*ν* C=O), 1,602 (*ν* C=C), 1,524 (*ν* NO_2_), 1,481 (*ν* C=C), 1,342 (*δ* C–H), 1,277 (*ν* C–N), 1,055 (*δ* C–H), 867, 849 (*ν* C–N), 721 (γ C–C) cm^−1^; ^1^H NMR (200 MHz, CDCl_3_): *δ* = 1.42 (d, 6H, 2 CH_3_, *J* = 6.6 Hz), 2.62 (s, 3H, SCH_3_), 3.66–3.75 (m, 1H, SCH), 8.01 (d, 2H, Ph, *J* = 8.7 Hz), 8.28–8.37 (m, 2H, Ph), 9.50 (s, 1H, NH) ppm; ^13^C NMR (50 MHz, CDCl_3_): *δ* = 15.34 (SCH_3_), 23.94 (CH_3_), 30.96 (CH), 124.09 (Ph), 128.36 (Ph), 142.31 (Ph-CO), 146.28 (Ph-NO_2_), 151.21 (C=N), 165.73 (C=O) ppm; MS (70 eV): *m*/*z* = 313.

#### *4*-*Nitrobenzoic acid 2*-*[(butylthio)(methylthio)methylene]hydrazide* (**20**, C_13_H_17_N_3_O_3_S_2_)

This compound was obtained as pale yellow crystals in 78% yield (0.33 g). M.p.: 60–62 °C; IR (KBr):$\bar{v}$ = 3,170 (*ν* N–H), 2,959, 2,928 (*ν* C–H), 1,672, 1,647 (*ν* C=O), 1,522 (*ν* NO_2_), 1,456 (*ν* C=C), 1,344 (*δ* C–H), 1,291, 864 (*ν* C–N), 721 (γ C–C) cm^−1^; ^1^H NMR (200 MHz, CDCl_3_): *δ* = 0.95 (t, 3H, CH_3_, *J* = 7.3 Hz), 1.35–1.57 (m, 2H, CH_2_), 1.62–1.89 (m, 2H, CH_2_), 2.60 (s, 1H, SCH_2_), 3.06 (t, 2H, SCH_2_, *J* = 6.8 Hz), 8.00 (d, 2H, Ph, *J* = 8.6 Hz), 8.19 (d, 2H, Ph, *J* = 8.5 Hz), 9.70 (s, 1H, NH) ppm; ^13^C NMR (50 MHz, CDCl_3_): *δ* = 14.18 (CH_3_), 15.21 (SCH_3_), 21.82 (CH_2_), 28.13 (SCH_2_), 32.57 (CH_2_), 124.58 (Ph), 128.14 (Ph), 142.91 (Ph-CO), 146.57 (Ph-NO_2_), 151.23 (C=N), 166.17 (C=O) ppm.

#### *4*-*Nitrobenzoic acid 2*-*[(decylthio)(methylthio)methylene]hydrazide* (**21**, C_19_H_29_N_3_O_3_S_2_)

This compound was obtained as a yellow solid in 77% yield (0.41 g). M.p.: 43–44 °C; IR (KBr):$\bar{v}$ = 3,181 (*ν* N–H), 2,921, 2,853 (*ν* C–H), 1,672, 1,647 (*ν* C=O), 1,523 (*ν* NO_2_), 1,464 (*ν* C=C), 1,344 (*δ* C–H), 863 (*ν* C–N), 720 (γ C–C) cm^−1^; ^1^H NMR (200 MHz, CDCl_3_): *δ* = 0.87 (t, 3H, CH_3_, *J* = 5.9 Hz), 1.26–1.46 (m, 12H, 6 CH_2_), 1.62–1.73 (m, 2H, CH_2_), 1.78–1.89 (m, 2H, CH_2_), 2.62 (s, 3H, SCH_3_), 3.06 (t, 2H, SCH_2_, *J* = 7 Hz), 8.00 (d, 2H, Ph, *J* = 8.8 Hz), 8.19 (d, 2H, Ph, *J* = 8.9 Hz), 9.37 (s, 1H, NH) ppm; ^13^C NMR (50 MHz, DMSO-*d*_*6*_): *δ* = 14.20 (CH_3_), 15.17 (SCH_3_), 22.36 (CH_2_), 28.20 (SCH_2_), 28.66 (CH_2_), 28.71 (CH_2_), 28.96 (CH_2_), 29.19 (CH_2_), 29.80 (CH_2_), 31.56 (CH_2_), 124.87 (Ph), 127.98 (Ph), 139.50 (Ph-CO), 149.35 (Ph-NO_2_), 155.17 (C=N), 164.28 (C=O) ppm.

#### *4*-*Nitrobenzoic acid 2*-*[(methylthio)(phenylmethylthio)methylene]hydrazide* (**22**, C_16_H_15_N_3_O_3_S_2_)

This compound was obtained as yellow flakes in 77% yield (0.36 g). M.p.: 124–125 °C; IR (KBr):$\bar{v}$ = 3,175 (*ν* N–H), 3,029, 2,999, 2,931 (*ν* C–H), 1,677, 1,651 (*ν* C=O), 1,600 (*ν* C=C), 1,535 (*ν* NO_2_), 1,342 (*δ* C–H), 1,288 (*ν* C–N), 1,071 (*δ* C–H), 865 (*ν* C–N), 719 (γ C–C) cm^−1^; ^1^H NMR (200 MHz, CDCl_3_): *δ* = 2.62 (s, 3H, SCH_3_), 4.19 (s, 2H, SCH_2_), 7.30–7.44 (m, 5H, Ph), 7.69–7.83 (m, 2H, Ph), 8.25 (d, 2H, Ph, *J* = 8.7 Hz), 9.25 (s, 1H, NH) ppm; ^13^C NMR (50 MHz, CDCl_3_): *δ* = 15.29 (SCH_3_), 37.81 (SCH_2_), 124.85 (Ph), 128.01 (Ph), 128.18 (Ph), 128.61 (Ph), 128.64 (Ph), 128.78 (Ph), 128.83 (Ph), 135.02 (Ph-CH_2_), 135.79 (Ph-CO), 148.47 (Ph-NO_2_), 153.43 (C=N), 165.97 (C=O) ppm.

#### Tuberculostatic assay

Investigations were performed by a classical test-tube method of a successive dilution in Youmans’ modification of Proskauer and Beck’s liquid medium containing 10% bovine serum [27, 28]. Bacterial suspensions were prepared from 14-day-old cultures of slowly growing strains and from 48-hour-old cultures of saprophytic strains [29, 30]. Solutions of the compounds in ethylene glycol were tested. Stock solutions contained 10 mg of the compounds in 1 cm^3^. Dilutions (in a geometric progression) were prepared in Youmans’ medium. Medium containing no investigated substances or containing isoniazid (INH) or pyrazinamide (PZA) as reference drugs were used for comparison. Dimethyl 4-nitrobenzoylcarbonohydrazonodithioate (**1**) described earlier [14] was also included in the tests. Incubation was performed at 37 °C. The MIC values were determined as the minimum concentration inhibiting the growth of the tested tuberculous strains in relation to the probe with no tested compound.

#### X-ray crystallography

Single crystals of compound **18** suitable for X-ray diffraction were obtained from ethanol by slow evaporation of the solvent at room temperature. X-ray data were collected on a Bruker SMART diffractometer with APEX II area detector. Data reduction was performed by means of the Bruker software SAINT (Version 6.45) and SADABS (Version 2.10) [31]. The structure was solved by direct methods using SHELXTL [32] and refined by full-matrix least squares [33]. Final coordinates and details of measurement, solution, and refinement have been deposited with the Cambridge Crystallographic Data Centre (no. 845158). All details of the crystal data may be obtained free of charge via http://www.ccdc.cam.ac.uk/conts/retrieving.html or from the Cambridge Crystallographic Data Centre, 12 Union Road, Cambridge CB2 1EZ, UK; fax: +44 1223 336033; or deposit@ccdc.cam.ac.uk.

## Electronic supplementary material

Below is the link to the electronic supplementary material.

Supplementary material 1 (JPEG 2256 kb)

Supplementary material 2 (JPEG 2415 kb)

Supplementary material 3 (JPEG 2181 kb)

Supplementary material 4 (TIFF 3871 kb)

Supplementary material 5 (TIFF 3669 kb)

Supplementary material 6 (TIFF 4822 kb)

Supplementary material 7 (TIFF 4503 kb)

Supplementary material 8 (TIFF 4057 kb)

Supplementary material 9 (TIFF 3992 kb)

Supplementary material 10 (TIFF 4356 kb)

Supplementary material 11 (TIFF 4368 kb)

Supplementary material 12 (TIFF 4347 kb)

Supplementary material 13 (TIFF 4245 kb)

Supplementary material 14 (TIFF 6102 kb)

Supplementary material 15 (TIFF 4328 kb)

Supplementary material 16 (TIFF 2556 kb)

Supplementary material 17 (TIFF 2065 kb)

Supplementary material 18 (TIFF 2719 kb)

Supplementary material 19 (TIFF 3613 kb)

Supplementary material 20 (TIFF 2104 kb)

Supplementary material 21 (TIFF 6269 kb)

Supplementary material 22 (TIFF 2383 kb)

Supplementary material 23 (TIFF 6120 kb)

Supplementary material 24 (TIFF 6791 kb)

Supplementary material 25 (TIFF 3936 kb)

Supplementary material 26 (TIFF 3065 kb)

Supplementary material 27 (TIFF 3221 kb)
