# Supplementary figures and images for: Synthesis, characterization, and tuberculostatic activity of novel 2-(4-nitrobenzoyl)hydrazinecarbodithioic acid derivatives
Source: Monatsh Chem. 2012 Jan 24;143(4):607–17. doi: 10.1007/s00706-011-0708-y (PMC4494774; doi:10.1007/s00706-011-0708-y)

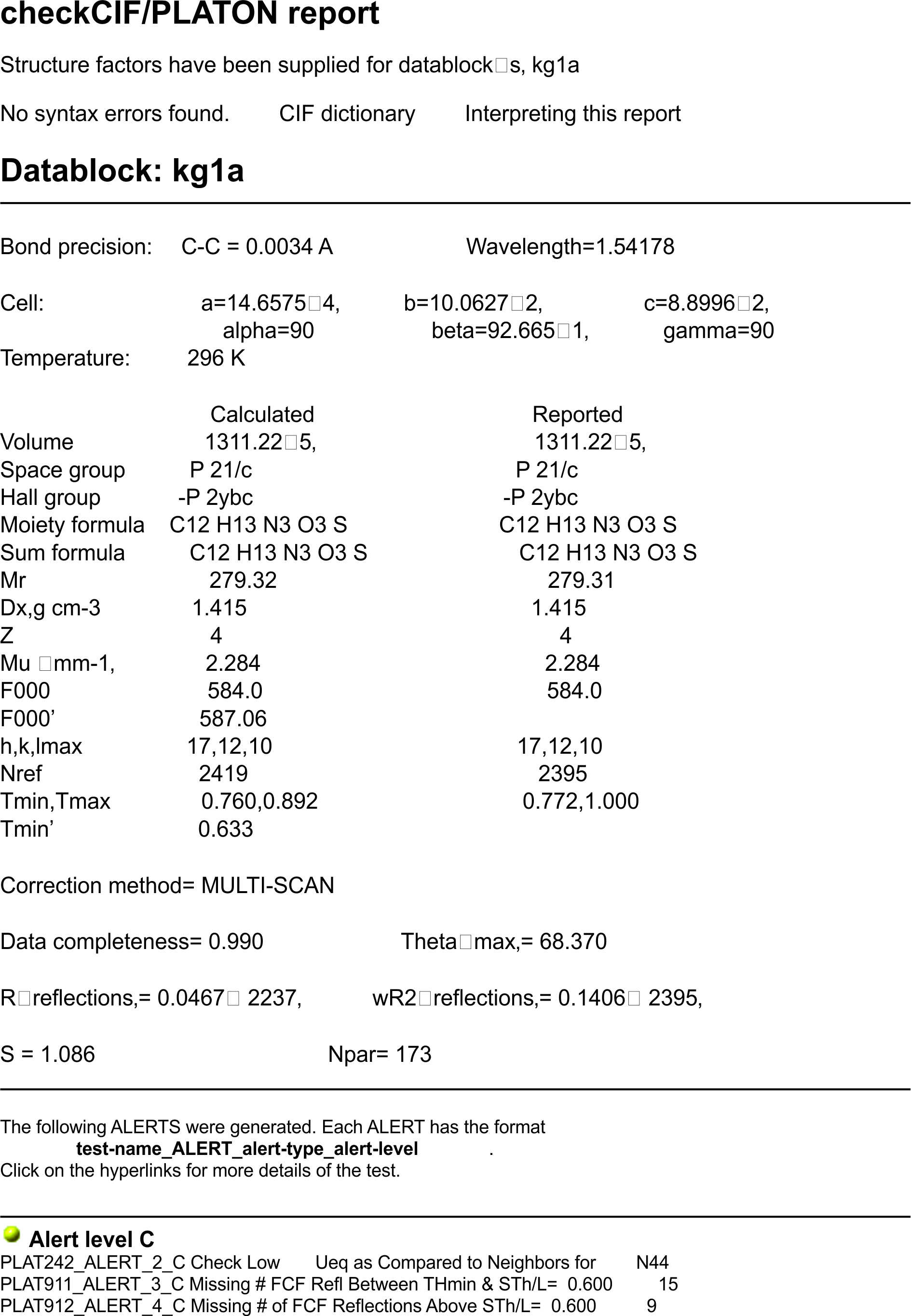

Supplement: Supplementary file 1 — Supplementary material 1 (JPEG 2256 kb) [file 706_2011_708_MOESM1_ESM.jpg]

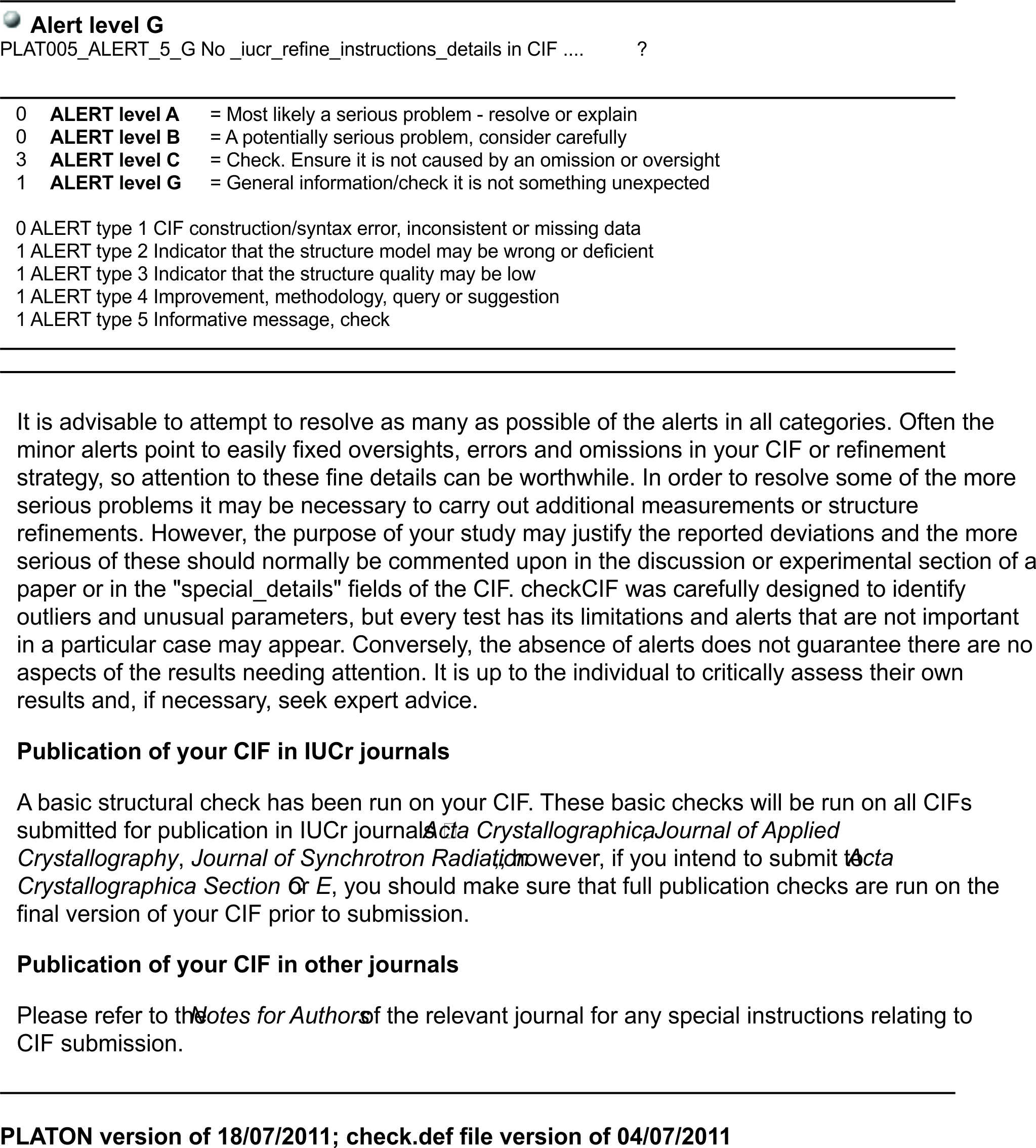

Supplement: Supplementary file 2 — Supplementary material 2 (JPEG 2415 kb) [file 706_2011_708_MOESM2_ESM.jpg]

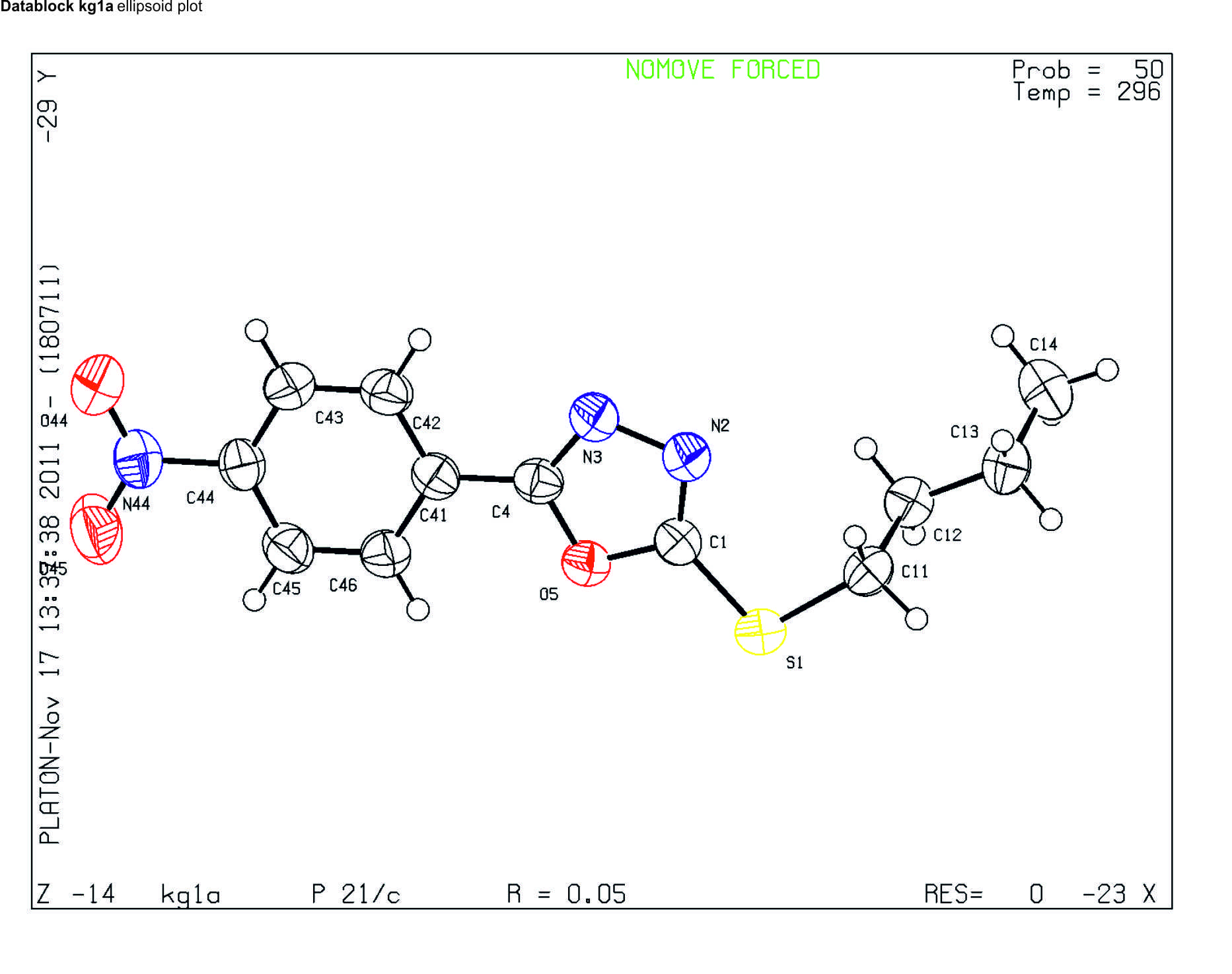

Supplement: Supplementary file 3 — Supplementary material 3 (JPEG 2181 kb) [file 706_2011_708_MOESM3_ESM.jpg]
